# Comparison between walking and moderate-to-vigorous physical activity: associations with metabolic syndrome components in Korean older adults

**DOI:** 10.4178/epih.e2020066

**Published:** 2020-10-28

**Authors:** Ki-Yong An

**Affiliations:** Faculty of Kinesiology, Sport, and Recreation, University of Alberta, Edmonton, AB, Canada

**Keywords:** Exercise, Metabolic syndrome, Walking, Aged

## Abstract

**OBJECTIVES:**

The purpose of this study was to compare moderate-to-vigorous physical activity (MVPA) with walking in terms of associations with metabolic syndrome components in Korean older adults.

**METHODS:**

Data on 1,388 older adults (age ≥65 years) from the Korea National Health and Nutrition Examination Survey 2018 were analyzed in this study. MVPA time and walking time per week were used as physical activity variables and blood pressure, waist circumference, glucose, high-density lipoprotein cholesterol, and triglyceride levels were analyzed as metabolic syndrome components. Partial correlations, analysis of covariance, and multinomial logistic regression were used for statistical analysis after adjusting for age, sex, smoking, and alcohol consumption.

**RESULTS:**

High-density lipoprotein cholesterol levels were significantly higher in the low MVPA/high walking and high MVPA/high walking groups than in the low MVPA/low walking group. Triglyceride levels were significantly lower in the high MVPA/high walking group than in the low MVPA/low walking and low MVPA/high walking groups. Engaging in <150 min/wk of MVPA increased the likelihood of abnormal blood pressure and metabolic syndrome by 1.81 times and 1.89 times, respectively, compared to ≥150 min/wk of MVPA. Engaging in <180 min/wk of walking raised the likelihood of having abnormal high-density lipoprotein levels by 1.32 times compared to ≥180 min/wk of walking.

**CONCLUSIONS:**

Not only MVPA but also walking was significantly associated with metabolic syndrome components in Korean older adults. Considering older adults’ preferences and exercise barriers, walking should be considered as an essential component of physical activity guidelines to prevent chronic diseases in older adults.

## INTRODUCTION

Metabolic syndrome is a complex metabolic disorder defined as a cluster of conditions that occur together, increasing the risk of heart disease, stroke, and type 2 diabetes. These conditions include excess abdominal obesity, hypertension, high fasting glucose, and abnormal cholesterol and triglyceride (TG) levels, and metabolic syndrome is diagnosed when a patient has 3 or more of those conditions. According to the Korea National Health and Nutrition Examination Survey (KNHANES) 2008-2013, the prevalence of metabolic syndrome increased with age in Korean adults; in particular, it was 6.8 times higher in male in their 70s and 35 times higher in female in their 70s than in male and female in their 20s, respectively [1].

Physical activity (PA) prevents obesity, type 2 diabetes, cardiovascular diseases, and metabolic syndrome, and improves the risk factors for those chronic diseases [2-6]. Accordingly, the World Health Organization (WHO) recommends at least 150 min/wk of moderate-intensity PA or at least 75 min/wk of vigorous-intensity PA, or an equivalent combination of moderate-intensity activity and vigorous-intensity activity. Moreover, for additional health benefits, twice the amount of PA is recommended [7]. However, older adults generally have lower levels of physical fitness; furthermore, they may face several physical barriers (e.g., arthritis and other chronic diseases) that hinder moderate physical activity (MPA) and vigorous. In a systematic review, Baert et al. [8] reported that specific health conditions, including poor balance, muscle weakness, and shortness of breath, as well as additional factors including lack of knowledge and fear of injury were barriers to exercise in older adults [8]. On the contrary, walking is a type of light-intensity PA that does not necessarily require a high-level of physical fitness or exercise knowledge, making it a straightforward option for many older adults. Additionally, walking can be done in everyday life as a mode of transportation even if people do not allocate extra time specifically for exercise. These factors may explain why walking is the most common form of exercise and has a significantly higher participation rate (68.2%) than other exercises such as bodyweight stretches and exercises (7.2%) and hiking (6.3%) among Korean older adults [9]. Previous studies have reported that walking has significant effects on reducing the risk of metabolic syndrome and improving various health conditions, despite being a low-intensity PA [10-12]. Nevertheless, the WHO PA guidelines mainly focus on MPA and VPA, and barely include walking or light PAs [7].

To date, it remains unclear whether moderate-intensity or vigorous-intensity PAs are essential to gain health benefits, as the WHO recommends, or whether walking may play a role as an alternative to moderate-intensity to vigorous-intensity PAs. Therefore, this study compared walking and moderate-intensity to vigorous-intensity PA in association with metabolic syndrome and its components in Korean older adults using the KNHANES 2018.

## MATERIALS AND METHODS

### Participants

Of a total of 7,992 participants in the KNHANES 2018, 1,388 older adults aged over 65 who completed assessments for PAs, the 5 components of metabolic syndrome, smoking status, and alcohol consumption were included in the analyses.

### Physical activity assessments

PA was assessed using the Korean version of the Global Physical Activity Questionnaire [13]. Leisure-time PA (min/wk) and total walking time (min/wk), including transportation but not occupational PA, were used as PA variables in this study considering that the participants were aged over 65. To calculate the total weighted moderate-to-vigorous physical activity (MVPA) time, VPA (8 metabolic equivalents [METs]) time was doubled and then added to MPA (4 METs) time based on METs of each PA. The total weighted MVPA was categorized into 2 groups (≥ 150 min/wk vs. < 150 min/wk) based on the WHO PA guidelines. Total walking time was considered as light PA (3.3 METs) and categorized into 2 groups (≥ 180 min/wk vs. < 180 min/wk) to generate an equivalent cut-off to the MVPA categorization based on total METs per week.

### Components of metabolic syndrome

The components of metabolic syndrome include waist circumference (WC), blood pressure (BP), fasting glucose, high-density lipoprotein cholesterol (HDL-C), and TG, which were assessed by trained research staff at mobile examination centers. We defined metabolic syndrome as the presence of 3 or more of the following components: (1) WC ≥ 90 cm for male and ≥ 80 cm for female; (2) systolic blood pressure (SBP) ≥ 130 mmHg or diastolic blood pressure (DBP) ≥ 85 mmHg; (3) fasting glucose > 100 mg/dL; (4) HDL-C < 40 mg/dL for male and < 50 mg/dL for female; and (5) TG ≥ 150 mg/dL.

### Statistical analysis

Partial correlations were used to examine the relationships between the PA variables and the 5 metabolic syndrome components. Analysis of covariance was used to examine differences in metabolic syndrome components among 4 groups defined according to the PA guidelines: (1) low MVPA/low walking; (2) low MVPA/high walking; (3) high MVPA/low walking; and (4) high MVPA/high walking. Lastly, multinomial logistic regression was used to examine the associations between meeting PA guidelines and meeting metabolic syndrome criteria. All analyses were performed after adjusting for age, sex, smoking status, and alcohol consumption using SPSS version 25.0 (IBM Corp., Armonk, NY, USA).

### Ethics statement

The KNHANES 2018 was approved by the Korea Centers for Disease Control and Prevention Institutional Review Board (2018-01-03-P-A).

## RESULTS

Participants’ characteristics are presented in Table 1. Briefly, 42.8% were male, the mean age of the participants was 72.7±5.0 years, and their mean body mass index was 24.3±3.0 kg/m^2^. It was found that 8.1% of the participants engaged in ≥ 150 min/wk of MVPA and 43.4% engaged in ≥ 180 min/wk of walking.

Table 2 shows the correlations between metabolic syndrome components and each PA after adjusting for age, sex, smoking status, and alcohol consumption. VPA was positively correlated with HDL-C (r=0.064) and negatively correlated with TG levels (r=-0.056). MPA was negatively correlated with SBP (r=-0.085) and TG (r=-0.068). MVPA had a positive correlation with HDL-C (r=0.073) and negative correlations with SBP (r=-0.061) and TG levels (r=-0.082). Walking was negatively correlated with TG levels (r=-0.065).

Table 3 shows differences in metabolic syndrome components according to MVPA and walking time after adjusting for age, sex, smoking status, and alcohol consumption. Participants were categorized into 2 groups based on whether they met the criterion of 150 min/wk of MVPA and then each group was categorized into 2 groups based on whether they engaged in 180 min/wk of walking. Finally, metabolic syndrome components were compared among the 4 groups. HDL-C levels were significantly higher in the low MVPA/high walking (48.9±0.5 mg/dL) and the high MVPA/high walking groups (49.9±1.3 mg/dL) than in the low MVPA/low walking group (47.2±0.4 mg/dL). TG levels were significantly lower in the high MVPA/high walking group (104.8±10.1 mg/dL) than in the low MVPA/low walking group (137.2±3.3 mg/dL) and the low MVPA/high walking group (127.2±3.9 mg/dL). There were no significant differences among groups in WC, SBP, DBP, or glucose levels.

Table 4 shows the likelihood of meeting metabolic syndrome criteria according to MVPA and walking time. If participants engaged in < 150 min/wk of MVPA, they were more likely to meet the BP criterion for metabolic syndrome (odds ratio [OR], 1.81; 95% confidence interval [CI], 1.18 to 2.77; p<0.01) and to have metabolic syndrome (OR, 1.89; 95% CI, 1.19 to 3.00; p<0.01) than those who engaged in ≥ 150 min/wk of MVPA. If participants engaged in < 180 min/wk of walking, they were more likely to meet the HDL-C criterion for metabolic syndrome (OR, 1.32; 95% CI, 1.04 to 1.66; p<0.05) than those who engaged in ≥ 180 min/wk of walking.

## DISCUSSION

This study compared the associations of walking and MVPA with metabolic syndrome components in Korean older adults. Not only MVPA but also walking was significantly associated with components of metabolic syndrome. HDL-C, in particular, was observed to be more significantly associated with walking than with MVPA.

Benefits of walking have been reported in several previous studies [10-12]. As expected, significant relationships between walking and metabolic syndrome components were observed in this study. More interestingly, our findings showed that the low MVPA/low walking group had significantly lower levels of HDL-C than the low MVPA/high walking group; however, no significant difference was found in comparison with the high MVPA/low walking group for any metabolic syndrome component, including HDL-C. These results suggest that even if Korean older adults do not engage in MVPA for more than 150 min/wk, if they walk at least 180 min/wk, they may be less likely to have metabolic syndrome. The regression analysis results also showed the beneficial role of walking, and in particular demonstrated that walking may reduce the risk of abnormal HDL-C levels. Nevertheless, to date, the importance of walking has been underestimated compared to MVPA. The WHO emphasizes MVPA not only for adults, but also for older adults. Although walking is mentioned along with the recommendations for increasing overall PA levels, there are no detailed guidelines on walking, unlike MVPA [7]. The importance of MVPA for health promotion and chronic disease prevention has been already well established [14-16], and the results of this study also support those findings. Thus, for individuals who have no health problems and have access to an appropriate environment to engage in MVPA, doing so would certainly be the best option. However, older adults with physical barriers due to poor health conditions, such as knee arthritis, cardiovascular diseases, overweight, and a low fitness level, may not find it possible to engage in VPA or even MPA. These trends were also observed in this study. As can be seen in Table 1, the percentage of Korean older adults engaging in more than 180 minutes of walking per week was 43.4%, but only 8.1% engaged in MVPA for more than 150 min/wk. Time spent in VPA, in particular, was less than 5 min/wk. Moreover, physical barriers are not the only reason why older adults have difficulties engaging in MVPA. A systematic review by Baert et al. [8] reported that a lack of knowledge and fear of injury functioned as barriers to PA participation among older adults in addition to loss of balance, weakness of muscle, shortness of breath, and overweight. As the intensity of exercise increases, the risk of injury increases accordingly; thus, in order to prevent this risk, older adults should be aware of appropriate exercise skills and exercise knowledge. However, older adults may be less likely to have highly accurate exercise knowledge.

In contrast, walking can be relatively easily performed by anyone without a high-risk of injury or great difficulties, and no additional cost or exercise equipment is required. This may be the reason why Korean older adults most strongly prefer walking as a leisure-time PA [9]. High doses and intensities of exercise are important, but reducing physical inactivity such as sedentary time and screen time, by light PA is another important component that we should consider for improving health conditions [17-19]. Furthermore, the amount of walking in this study was the total amount of walking, including walking for transportation as well as walking for exercise. Therefore, the findings of this study suggest that even if individuals do not exercise deliberately, increasing the amount of walking in their everyday lives may also improve components of metabolic syndrome. Therefore, walking may be suitable both for older adults who cannot exercise due to health conditions and for elderly individuals who cannot exercise due to a lack of time or lack of information.

Currently, there are no official PA or exercise guidelines developed by the government in Korea; therefore, the WHO PA recommendations [7] were used in this study. The WHO PA recommendations are among the most widely used PA guidelines worldwide, but the required doses and types of exercise may slightly differ according to race/ethnicity or region. For these reasons, several countries have developed and used their own specific PA guidelines. In this regard, Korea may also need to develop and disseminate PA or exercise guidelines by age groups based on specific characteristics of the Korean populations. Furthermore, if Korea-specific PA guidelines are successfully used, this may provide positive outcomes in an aging society in terms of promoting health, reducing medical expenses, and increasing the working population.

This study used cross-sectional data; thus, causal relationships between metabolic syndrome components and walking or MVPA could not be clearly explained. Furthermore, self-reported PA may be another limitation of the study. However, this study is novel in terms of the nationally-representative dataset, a relatively large sample size of 1,388, the analysis of the most recently collected and available data (i.e., a survey done in 2018), and the research objective of comparing walking and MVPA. Future research should examine the effects of different intensities of PA on the components of metabolic syndrome using randomized controlled designs. Moreover, although this study used 180 minutes as the criterion for walking, research to determine the optimal amount of walking is warranted.

This study suggests that not only MVPA but also walking is significantly associated with the prevention and improvement of metabolic syndrome in Korean older adults. Considering the barriers to exercise and exercise-related preferences among older adults, walking should be considered as an essential component of PA guidelines for older adults to prevent chronic diseases in Korea.

## Figures and Tables

**Table 1.** Participants’ characteristics

Variables	Total (n=1,388)	Male (n=594)	Female (n=794)
Age (yr)	72.7±5.0	72.6±5.0	72.7±5.0
Weight (kg)	61.0±9.8	66.4±9.1	57.0±8.3
Body mass index (kg/m^2^)	24.3±3.0	24.0±2.8	24.5±3.2
WC (cm)	85.6±8.9	87.8±8.8	84.0±8.5
SBP (mmHg)	128.9±17.5	127.2±17.4	130.2±17.6
DBP (mmHg)	72.7±10.0	72.2±10.0	73.0±10.0
Glucose (mg/dL)	107.9±24.8	110.1±26.4	106.2±23.4
HDL-C (mg/dL)	48.0±11.5	46.1±10.8	49.5±11.8
Triglyceride (mg/dL)	131.5±89.8	131.1±78.5	131.8±97.4
VPA (min/week)	4.7±40.8	9.7±60.4	0.9±12.1
MPA (min/wk)	27.0±93.3	41.4±114.4	16.3±71.9
Walking (min/wk)	229.5±332.6	267.7±331.0	200.9±331.2
MVPA ≥150 min/wk	112 (8.1)	78 (13.1)	34 (4.3)
Walking ≥180 min/wk	603 (43.4)	295 (49.7)	308 (38.8)

Values are presented as mean±standard deviation or number (%). WC, waist circumference; SBP, systolic blood pressure; DBP, diastolic blood pressure; HDL-C, high-density lipoprotein cholesterol; VPA, vigorous physical activity; MPA, moderate physical activity; MVPA, moderate-to-vigorous physical activity.

**Table 2.** Correlations between different intensities of physical activity and metabolic syndrome components^1^

Variables	WC	SBP	DBP	Glucose	HDL-C	TG
VPA	-0.010	-0.001	0.010	-0.017	0.064^*^	-0.056^*^
MPA	-0.050	-0.085^**^	-0.048	-0.043	0.047	-0.068^*^
MVPA	-0.041	-0.061^*^	-0.028	-0.040	0.073^**^	-0.082^**^
Walking	-0.005	-0.006	0.014	-0.027	0.051	-0.065^*^

WC, waist circumference; SBP, systolic blood pressure; DBP, diastolic blood pressure; HDL-C, high-density lipoprotein cholesterol; TG, triglyceride; VPA, vigorous physical activity; MPA, moderate physical activity; MVPA, moderate-to-vigorous physical activity.

1 Adjusted for age, sex, alcohol consumption, and smoking status.

* p<0.05,

** p<0.01.

**Table 3.** Comparison of the associations of MVPA and walking with metabolic syndrome components^1^

Variables	Low MVPA		High MVPA	
	Low walking (n=755)	High walking (n=521)	Low walking (n=30)	High walking (n=82)
WC (cm)	85.9±0.3	85.5±0.4	83.7±1.6	84.1±1.0
SBP (mmHg)	129.1±0.6	129.5±0.8	124.4±3.2	125.6±2.0
DBP (mmHg)	72.6±0.4	72.9±0.4	70.4±1.8	72.5±1.1
Glucose (mg/dL)	109.3±0.9	106.6±1.1	103.8±4.5	103.6±2.8
HDL-C (mg/dL)	47.2±0.4	48.9±0.5^*^	48.4±2.1	49.9±1.3^*^
TG (mg/dL)	137.2±3.3	127.2±3.9	134.0±16.4	104.8±10.1^*^^

Values are presented as estimated mean±standard error. WC, waist circumference; SBP, systolic blood pressure; DBP, diastolic blood pressure; HDL-C, high-density lipoprotein cholesterol; TG, triglyceride; MVPA, moderate-to-vigorous physical activity.

1 Adjusted for age, sex, alcohol consumption, and smoking status.

* p<0.05 vs. low MVPA/low walking;

^ p<0.05 vs. low MVPA/high walking.

**Table 4.** Multinomial logistic regression model estimating the likelihood of meeting the diagnostic criteria of metabolic syndrome according to MVPA and walking^1^

Variables (meeting vs. not meeting diagnostic criteria)^2^	MVPA		Walking	
	<150 min vs. ≥150 min		<180 min vs. ≥180 min	
	OR (95% CI)	p-value	OR (95% CI)	p-value
WC (≥90 cm for male/≥80 cm for female)	1.35 (0.89, 2.06)	0.16	1.10 (0.87, 1.38)	0.44
BP (SBP ≥130 mmHg or DBP≥85 mmHg)	1.81 (1.18, 2.77)	<0.01	0.99 (0.80, 1.24)	0.95
Glucose (>100 mg/dL)	1.35 (0.90, 2.04)	0.15	1.02 (0.82, 1.27)	0.86
HDL-C (<40 mg/dL for male/ <50 mg/dL for female)	1.14 (0.72, 1.79)	0.57	1.32 (1.04, 1.66)	0.02
TG (≥150 mg/dL)	1.54 (0.94, 2.53)	0.09	1.17 (0.91, 1.50)	0.22
Metabolic syndrome (≥3 components)	1.89 (1.19, 3.00)	<0.01	1.17 (0.93, 1.47)	0.17

OR, odds ratio; CI, confidence interval; WC, waist circumference; BP, blood pressure; SBP, systolic blood pressure; DBP, diastolic blood pressure; HDL-C, high-density lipoprotein cholesterol; TG, triglyceride; MVPA, moderate-to-vigorous physical activity.

1 Adjusted for age, sex, alcohol consumption, smoking status, walking (for MVPA), and MVPA (for walking).

2 The number of participants meeting the diagnostic criteria was 775 for WC, 660 for BP, 724 for glucose, 607 for HDL-C, 395 for TG, and 589 for metabolic syndrome out of a total of 1,388.

## SUPPLEMENTARY MATERIALS

Korean version is available at http://www.e-epih.org/.
