# Supplementary material for: Comparison between walking and moderate-to-vigorous physical activity: associations with metabolic syndrome components in Korean older adults
Source: Epidemiol Health. 2020 Oct 28;42:e2020066. doi: 10.4178/epih.e2020066 (PMC7871155; doi:10.4178/epih.e2020066)
Supplement: Supplementary file 1 [file epih-42-e2020066-suppl.docx]

**한국 노인들의 걷기와 중고강도 신체활동 간의 비교연구: 대사증후군 위험요인과의 관계를 중심으로**

**Comparison between walking and moderate-to-vigorous physical activity: associations with metabolic syndrome components in Korean older adults**

안기용 (ORCID: 0000-0001-7529-1314)

Ki-Yong An

Faculty of Kinesiology, Sport, and Recreation, University of Alberta, Canada

**Running title:** Associations of walking and moderate-to-vigorous physical activity with metabolic syndrome

**Corresponding author**: Ki-Yong An, PhD, 1-115 University Hall, University of Alberta, 116St. and 85Ave., Edmonton, Alberta, Canada T6G 2H9, kiyong1@ualberta.ca

**ABSTRACT**

**Objectives**: The purpose of this study was to compare moderate-to-vigorous physical activity (MVPA) with walking in terms of associations with metabolic syndrome components in Korean older adults.

**Methods**: Data on 1,388 older adults (age ≥65 years) from the Korea National Health and Nutrition Examination Survey 2018 were analyzed in this study. MVPA time and walking time per week were used as physical activity variables and blood pressure, waist circumference, glucose, high-density lipoprotein, and triglyceride levels were analyzed as metabolic syndrome components. Partial correlations, analysis of covariance, and multinomial logistic regression were used for statistical analysis after adjusting for age, sex, smoking, and alcohol consumption.

**Results**: High-density lipoprotein levels were significantly higher in the low MVPA/high walking and high MVPA/high walking groups than in the low MVPA/low walking group. Triglyceride levels were significantly lower in the high MVPA/high walking group than in the low MVPA/low walking and low MVPA/high walking groups. Engaging in <150 min/wk of MVPA increased the likelihood of abnormal blood pressure and metabolic syndrome by 1.8 times and 1.9 times, respectively, compared to ≥150 min/wk of MVPA. Engaging in <180 min/wk of walking raised the likelihood of having abnormal high-density lipoprotein levels by 1.3 times compared to ≥180 min/wk of walking.

**Conclusion**: Not only MVPA but also walking was significantly associated with metabolic syndrome components in Korean older adults. Considering older adults’ preferences and exercise barriers, walking should be considered as an essential component of physical activity guidelines to prevent chronic diseases in older adults.

**Keywords**: metabolic syndrome, walking, exercise, aged

**INTRODUCTION**

대사증후군은 당뇨, 심혈관 질환, 고지혈증 등 다양한 만성질환의 증상들이 동시에 나타나는 것을 의미한다. 대사증후군의 진단은 허리둘레, 혈압, 공복시 혈당, 고밀도 지단백 콜레스테롤, 중성지방의 다섯 가지 지표를 통해서 결정되며, 이 중 세 가지 이상이 기준치를 넘을 경우 대사증후군으로 진단한다. 국민건강영양조사 자료를 이용한 선행 연구에 따르면, 우리나라 성인의 대사증후군 유병률은 나이가 들수록 증가하는 경향을 보였으며, 20대에 비해 70대 이상에서 남성에서는 6.8배, 여성에서는 무려 35배의 증가율을 보였다  [1].

한편, 신체활동과 운동의 참여는 비만, 당뇨, 심혈관 질환은 물론 대사증후군의 예방 및 예후에 긍정적인 영향을 미친다고 잘 알려져 있다  [2-6]. 이에 따라 세계보건기구에서는 65세 이상 노인들에게 주당 최소 150분 이상의 중강도 또는 75분 이상의 고강도 신체활동, 또는 같은 양의 중, 고강도 신체활동의 적절한 조합을 권고하고 있으며, 추가적인 건강상의 이득을 위해서는 그 두 배에 해당하는 주당 중강도 300분, 고강도 150 이상의 신체활동 참여를 권고하고 있다 [7]. 하지만 노인들은 일반 성인에 비해 상대적으로 체력수준이 낮고 특히, 만성질환 등 건강상의 문제가 있는 경우, 중, 고강도 신체활동을 참여하는 데에 제한점이 있다. Baert 등의 체계적 문헌고찰에 따르면 노인들은 운동참여에 대한 장애물로 균형감각의 상실, 근육약화, 호흡의 짧아짐, 과체중 등 주로 신체적인 기능의 변화를 꼽았으며, 신체기능의 변화 외에도 운동에 대한 지식의 부족, 부상에 대한 두려움 등을 부가적인 운동참여의 제약요인으로 생각하고 있었다  [8].

하지만 걷기는 높은 체력수준이나 운동에 대한 지식을 필요로 하지 않아 노인들도 쉽게 참여할 수 있는 저강도 운동이며, 특별히 시간을 내지 않더라도 일상생활 속에서 실천할 수 있는 신체활동이기도 하다. 실제로 걷기는 우리나라 노인들이 가장 즐겨하는 운동으로, 체조 (7.2%)나 등산 (6.3%) 등 다른 운동과 비교해 월등하게 높은 참여율 (68.2%) 을 보이고 있다  [9]. 이러한 걷기는 저강도 신체활동임에도 불구하고, 노인들의 대사증후군 위험을 낮추고, 건강을 증진시키는데 큰 효과가 있는 것으로 여러 연구들을 통해 보고되고 있다  [10-12]. 하지만, 위에서 언급한 바와 같이 세계보건기구에서 권고하는 운동지침은 중,고강도 신체활동에 초점이 맞춰져 있고, 걷기에 대한 내용은 거의 포함되어 있지 않다  [7].

현재까지 노인들에게서 걷기가 중,고강도 신체활동 만큼 건강상의 이득을 제공할 수 있는지, 세계보건기구에서 권고하는 대로 중,고강도 신체활동을 꼭 포함시켜야만 건강상의 이득을 얻을 수 있는 것인지에 대한 과학적 근거는 아직 미흡하다. 따라서, 본 연구에서는 국민건강영양조사 2018년 자료를 사용하여, 한국 노인들에게서 대사증후군의 다섯 가지 지표를 이용하여, 걷기와 중,고강도 신체활동과의 관계를 비교 분석하였다.

**METHODS**

**Participants**

본 연구는 2018년 국민건강영양조사에 참여한 7,992명의 대상자 중 중, 고강도 신체활동, 총 걷기, 다섯가지 대사증후군 요인 및 흡연과 음주량에 대해 모든 설문과 측정을 완료한 만 65세 이상의 노인 총 1,388명을 대상으로 실시되었다. 국민건강 영양조사는 생명윤리법 제 2조 제1호 및 동법 시행규칙 제2조 제2항 제1호에 따라 국가가 직접 공공복리를 위해 수행하는 연구에 해당하여 2015년부터 연구윤리심의위원회의 심의를 받지 않고 수행되고 있다.

**Physical activity assessment**

국민건강영양조사에서는 Global Physical Activity Questionnaire (GPAQ)의 한국어 버전을 이용하여 신체활동량을 조사하였다  [13]. 본 연구에서는 대상자가 65세 이상의 노인임을 고려하여 신체활동 변인으로 일 관련 신체활동을 제외하고, 주당 여가시간 중강도 및 고강도 신체활동 시간 (분) 과, 장소 이동과 관련된 걷기를 포함한 총 걷기시간 (분)을 사용하였다. 주당 중강도와 고강도 신체활동 시간의 합은 대사당량 (MET: metabolic equivalent)을 고려하여, 고강도 신체활동 (8 METs) 시간을 두배로 환산한 뒤 중강도 신체활동 (4METs) 시간과 합산한 값을 사용하였다. 또한, 신체활동 실천의 명목형 변수를 만들기 위해, 세계보건기구 운동지침에 따라 중, 고강도 신체활동 시간의 합산을 주당150분을 기준으로 두 그룹으로 나누어 사용하였다. 걷기시간의 명목형 변수는 대사당량을 고려하여, 중강도 신체활동 150분과 대등한 운동량을 충족하기 위해 걷기를 저강도 신체활동 (3.3 METs) 으로 간주하고, 주당 180분을 기준으로 하여 두 그룹으로 나누어 사용하였다.

**Components of the metabolic syndrome**

대사증후군 결정요인으로 허리둘레, 수축기 및 이완기 혈압, 혈당, 고밀도 지단백 콜레스테롤, 중성지방을 사용하였으며, 모든 대사증후군 변인은 이동검진센터에서 직접계측 방법을 이용한 검진조사를 통해 이루어졌다. 대사증후군 결정을 위한 기준은 다음과 같다: 1) 허리둘레: 남자는 90cm이상, 여자는 80cm 이상, 2)혈압: 수축기 혈압 130mmHg 이상 또는 이완기 혈압 85mmHg 이상, 3) 공복시 혈당: 100mg/dl 초과, 4) 고밀도 지단백 콜레스테롤: 남자는 40mg/dl 미만, 여자는 50mg/dl 미만, 5) 중성지방: 150mg/dl이상.

**Statistical analysis**

중강도, 고강도, 중,고강도 신체활동량, 걷기량과 다섯 가지 대사증후군 결정요인과의 상관관계는 편상관분석을 사용하여 분석하였다. 공분산분석을 위해, 중,고강도 신체활동을 주당 150분 기준으로 두 그룹으로 나누고, 각 그룹을 주당 걷기량 180분을 기준으로 다시 두 그룹으로 나누어, 중, 고강도 신체활동과 걷기량에 따라 네 그룹으로 구분하였다: 1) 중고강도 신체활동 낮음 /걷기 낮음, 2) 중고강도 신체활동 낮음/ 걷기 높음, 3) 중고강도 신체활동 높음/ 걷기 낮음, 4) 중고강도 신체활동 높음/ 걷기 높음. 공분산분석을 이용하여 네 그룹 간의 대사증후군 지표의 차이를 비교하였다. 마지막으로 다항 로지스틱 회귀분석을 이용하여 중,고강도 신체활동 주당 150분과 걷기 주당 180분을 충족하지 못했을 때, 각 대사증후군 지표들이 대사증후군 유무 구분 조건을 충족할 오즈비를 분석하였다. 모든 통계방법은 나이와 성별, 흡연여부와 음주량을 통제한 후 수행되었다. 통계분석을 위해서는 SPSS 25.0이 사용되었다.

**RESULTS**

연구 대상자들의 특성은 Table 1에 제시되어 있다. 전체 대상 자 중 남성의 비율은 약 42.8% 였고, 전체 대상자의 평균 연령은 72.7±5.0세, 평균 체질량지수는 24.3±3.0 kg/m^2^ 이었다. 전체 대상자 중 중고강도 신체활동을 주당 150분 이상 실천하는 대상자의 비율은 8.1%, 걷기를 주당 180분 이상 실천하는 대상자의 비율은 43.4%였다.

Table 2는 나이, 성별, 흡연유무, 음주량을 통제한 후 대사증후군의 다섯 가지 지표와 각 신체활동과의 상관관계를 보여준다. 고강도 신체활동은 고밀도 지단백 콜레스테롤과 양의 상관관계를 (r=0.064), 중성지방과 음의 상관관계를 (r=-0.056) 나타냈으며, 중강도 신체활동은 수축기 혈압 (r=-0.085)과 중성지방 (r=-0.068)과 음의 상관관계를 나타냈다. 또한, 중고강도 신체활동은 고밀도 지단백 콜레스테롤과 양의 상관관계를(r=0.073), 수축기 혈압과 (r=-0.061), 중성지방 (r=-0.082) 과는 음의 상관관계를 나타냈다. 걷기는 중성지방과 음의 상관관계 (r=-0.065)를 나타냈다.

Table 3은 나이, 성별, 흡연유무, 음주량을 통제한 후 중고강도 신체활동 및 걷기양에 따른 대사증후군의 다섯 가지 지표의 차이를 보여준다. 중고강도 신체활동량과 걷기량의 그룹을 나누기 위해 중고강도 신체활동량은 주당 150분을 기준으로, 걷기량은 주당 180분을 기준으로 각각 두 그룹으로 나누어, 중고강도 신체활동량과 걷기량이 모두 낮은 그룹, 중고강도 신체활동량만 높은 그룹, 걷기량만 높은 그룹, 두 가지 모두 높은 그룹으로 나누어 분석을 실시했다. 허리둘레, SBP, DBP, glucose에선 네 그룹간의 유의한 차이가 나타나지 않았다. 하지만 중고강도 신체활동량과 걷기양 모두 낮은 그룹에 비해, 중고강도 신체활동량이 낮고 걷기양만 높은 그룹에서 고밀도 지단백 콜레스테롤이 유의하게 높은 것으로 나타났다 (48.9±0.5 vs. 47.2±0.4 mg/dl). 또한, 중고강도 신체활동량과 걷기양 모두 낮은 그룹에 비해 중고강도 신체활동량과 걷기양 모두 높은 그룹에서 고밀도 지단백 콜레스테롤은 통계적으로 유의하게 높고 (49.9±1.3 vs. 47.2±0.4 mg/dl) 중성지방은 낮은 것으로 나타났다 (104.8±10.1 vs. 137.2±3.3 mg/dl). 또한, 중고강도 신체활동량은 낮고 걷기양만 높은 그룹에 비해 중고강도 신체활동과 걷기양이 모두 높은 그룹에서 중성지방이 유의하게 낮게 나타났다 (104.8±10.1 vs. 127.2±3.9 mg/dl).

Table 4는 나이, 성별, 흡연유무, 음주량을 통제한 후 중고강도 신체활동과 걷기양에 따라 대사증후군 다섯가지 지표들이 기준을 충족할 가능성 및 대사증후군 유병 가능성을 보여준다. 중고강도 신체활동을 주당 150분 이상 실천했을 때에 비해, 150분 미만으로 실천했을 경우 혈압이 기준이상으로 높을 확률이 1.8배 (p=0.007), 대사증후군 유병 가능성이 1.9배 (p=0.007) 증가하는 것으로 나타났다. 걷기를 주당 180분 이상으로 실천했을 때에 비해, 180분 미만으로 실천할 경우 고밀도 지단백 콜레스테롤이 기준 이하로 낮을 확률이 1.3배 (p=0.021) 증가하는 것으로 나타났다.

**DISCUSSION**

본 연구는 한국의 노인들을 대상으로 대사증후군 지표와의 관계에 대해 걷기와 중,고강도 신체활동을 비교분석 하였다. 분석결과, 중고강도 신체활동이 대사증후군 지표 및 대사증후군 유병과 유의한 관계가 있었으며, 걷기도 중,고강도 신체활동 못지않게 대사증후군 지표들과 유의한 관계가 있는 것으로 나타났다. 특히, 고밀도 지단백 콜레스테롤과는 걷기가 중,고강도 신체활동보다 더 밀접한 관계가 있는 것으로 관찰되었다.

걷기의 효과는 본 연구 이전에 많은 연구에서 이미 보고되어 왔다  [10-12]. 본 연구에서도 예상했던 대로 걷기와 대사증후군 지표들과의 유의한 관계가 관찰되었다. 심지어 공분산분석 결과에서는, 걷기와 중,고강도 신체활동량 모두 낮은 그룹이 중고강도 신체활동은 낮지만 걷기량이 높은 그룹보다 고밀도 지단백 콜레스테롤이 유의하게 낮게 나타났지만, 걷기량이 낮고, 중고강도 신체활동이 높은 그룹과는 고밀도 지단백 콜레스테롤을 포함한 어떤 대사증후군 지표에서도 유의한 차이가 나타나지 않았다. 본 결과는 우리나라 노인들에게서 중,고강도 신체활동을 주당 150분 이상 하지 않더라도 걷기를 주당 180분 실천한다면, 중,고강도 신체활동만 실천하고 걷기를 하지 않는 것보다 대사증후군 발병위험성을 더 낮출 수도 있다는 것을 시사한다. 회귀분석 결과에서도, 중고강도 신체활동이 혈압과 대사증후군 유병을 낮출 수 있음을 보여줌과 동시에 걷기를 통해 HDL을 정상수준으로 유지할 수 있음을 동시에 보여주고 있다. 그럼에도 불구하고, 지금까지 걷기는 중,고강도 신체활동에 비해 그 중요성이 과소평가 되어 왔다. 세계보건기구에서는 성인 뿐 아니라 노인들에게도 중,고강도 신체활동을 강조하고 있다. 전체적인 신체활동량의 증가를 권고하며 걷기에 대한 언급이 되어 있기는 하지만 중, 고강도 신체활동처럼 자세한 운동량에 대한 지침은 없다 [7]. 물론, 건강증진 및 만성질환 예방을 위한 중,고강도 운동의 중요성은 이미 너무 잘 알려져 있으며 [14-16], 본 연구에서도 그 중요성을 다시 한 번 확인하였다. 따라서, 건강상의 문제가 없고, 중,고강도 신체활동을 실천하기에 알맞은 조건을 갖추고 있다면 중,고강도 신체활동을 실천하는 것이 최고의 선택일 것이다. 하지만, 중, 고강도 신체활동의 참여가 어려운 노인들, 예를 들어, 무릎 관절염, 심장질환, 과체중, 낮은 체력 등 신체적인 제약이 있는 노인들에게는 고강도 운동은 거의 실천이 불가능하고, 중강도 운동도 일주일에 150분 이상 실천하기가 쉽지 않을 것이다. 이는 본 연구결과에서도 간접적으로 보여주고 있다. Table 1에서도 볼 수 있듯이, 우리나라 노인들 중 걷기를 주당 180분 이상 실천하는 비율은 43.4% 이지만, 중,고강도 신체활동을 주당 150분 이상 실철하는 비율은 8.1% 에 그치고 있다. 특히, 고강도 신체활동의 주당 실천시간은 5분에도 미치지 못한다. 노인들이 중,고강도 신체활동에 참여하기 어려운 이유는 신체적인 제약 뿐만이 아니다. Baert 등의 체계적 문헌고찰은 균형감각의 상실, 근육의 약화, 짧아진 호흡, 과체중 뿐 아니라 운동에 대한 지식부족, 부상에 대한 두려움도 노인들의 운동참여 제약요인으로 보고하였다 [8]. 운동의 강도가 증가하면 부상의 위험도 따라서 증가하고, 이를 예방하기 위해서는 그만큼 정확한 자세와 운동지식을 습득하고 있어야 한다. 하지만 노인들이 이런 운동지식을 갖추고 중,고강도 신체활동에 참여하는 경우는 많지 않을 것이다.

반면, 걷기는 남녀노소 누구나 쉽게 할 수 있어 진입장벽이 매우 낮으며, 부상의 위험도 적고, 추가 비용이나 운동기구가 필요하지 않다. 아마도 이런 이유 때문에 한국 노인들이 걷기를 가장 많이 선호하고 있을 것이라 사료된다 [9]. 중, 고강도 신체활동으로 운동량과 운동강도를 늘리는 것도 중요하지만, 걷기를 통해 좌식시간, 스크린 타임 등 신체비활동을 줄이는 것도 건강증진을 위해 매우 중요한 요인이다 [17-19]. 게다가 본 연구에서 사용된 걷기량은 운동으로써의 걷기 뿐 아니라 이동수단을 위한 걷기가 포함된 총 걷기량이다. 따라서, 본 연구결과는 꼭 시간을 내서 하는 운동이 아닌, 생활 속 신체활동으로써의 걷기량을 늘리는 것도 대사지표의 개선에 도움을 줄 수 있음을 시사한다. 이런 점을 고려한다면, 걷기는 건강상의 제약때문에 운동을 못하는 노인들 뿐 아니라, 시간부족, 정보부족의 이유로 운동에 참여하지 못하는 노인들에게도 생활 속 신체활동을 통해 건강을 증진시킬 수 위한 좋은 도구로 사용될 수 있을 것이다.

현재 한국에는 정부 차원에서 개발된 신체활동 또는 운동 지침이 없다. 따라서 본 연구에서는 중,고강도 신체활동량 충족기준으로 세계보건기구의 신체활동 지침 [7]을 사용하였다. 세계보건기구의 신체활동지침은 전세계적으로 가장 보편적으로 사용되고 있는 신체활동 지침이지만, 인종이나 지역적 특징에 따라 필요한 운동종류와 운동량은 조금씩 달라질 수 있다. 이러한 이유로 많은 나라들이 각 나라에 알맞은 고유의 신체활동 가이드라인을 개발하여 이용하고 있다. 한국도 한국 국민들의 특성을 고려한 연령별 신체활동 또는 운동 지침의 개발 및 보급이 필요하며, 신체활동 지침이 성공적으로 활용된다면 점점 고령화가 심각해지는 이 시점에 국민건강 증진, 의료비 지출 감소, 노동 인구의 증가 등 다양한 효과를 기대해 볼 수 있을 것이다.

본 연구는 횡단연구 자료를 사용했기 때문에 걷기나 중,고강도 신체활동과 대사지표 사이에 인과관계를 설명할 순 없었다. 또한, 대상자가 스스로의 신체활동량을 회상하여 기록하는 방법을 사용하였기 때문에 객관적인 신체활동 측정량에 비해서는 그 신뢰도가 낮을 수 있다. 하지만 1,388명이라는 상대적으로 많은 대상자 수와 국가 대표자료인 국민건강영양조사 자료를 사용했다는 점, 그 중에서도 가장 최근 조사된 2018년 자료를 사용했다는 점, 걷기와 중,고강도 신체활동을 비교하였다는 점에서 선행 연구들 과의 차별성 및 독창성이 있다. 향후에는 무작위 배정연구 또는 전향적 연구를 통해 걷기와 중,고강도 신체활동이 대사지표에 미치는 영향에 대해 조사하는 연구가 필요하다. 또한, 본 연구에서는 대사당량을 고려하여, 편의상 걷기의 기준을 180분으로 지정하여 분석하였지만, 과학적 근거에 기반하여 적당한 걷기 권장량을 조사하는 연구도 필요하다.

본 연구를 통해 한국 노인들에게서 중, 고강도 신체활동 뿐 아니라 걷기량의 증가도 대사증후군의 예방 및 개선과 매우 밀접한 연관이 있음을 알 수 있었다. 노인들의 신체적 제약 및 운동선호도를 고려하였을 때, 걷기를 포함한 노인들을 위한 현실적인 신체활동 가이드라인의 개발과 보급은 노인들의 신체활동 증진과 만성질환 예방에 큰 역할을 할 것이다.

**Conflict of interest**

The author has no conflicts of interest to declare for this study.

**Acknowledgement**

N/A

**Author contributions**

Conceptualization, Methodology, Formal analysis, Data curation, Writing- Original draft, Writing- Reviewing and Editing: KA

Funding acquisition, project administration, visualization: N/A

**Key message**

현재까지 저강도 신체활동 (걷기)은 중고강도 신체활동에 비해 상대적으로 그 중요성이 간과되어 왔다. 하지만 본 연구는 저강도 신체활동도 중고강도 신체활동 못지 않게 노인들에게서 대사증후군을 예방할 수 있는 중요한 역할을 할 수 있음을 보여준다. 게다가 노인들의 신체적 제약, 부상의 위험, 운동 선호도 등을 감안하면 걷기와 같은 저강도 신체활동은 노인들의 건강유지를 위한 신체활동 권고사항의 필수요소로 고려되어야 함을 제안한다.

**REFERENCES**

1.Tran BT, Jeong BY, Oh JK. The prevalence trend of metabolic syndrome and its components and risk factors in Korean adults: results from the Korean National Health and Nutrition Examination Survey 2008-2013. BMC Public Health 2017;17:71.

2.Kang DW, Lee EY, An KY, Min J, Jeon JY, Courneya KS. Associations between physical activity and comorbidities in Korean cancer survivors. Journal of Cancer Survivorship 2018;12:441-449.

3.Kim SH, Lee SH, Ahn KY, Lee DH, Suh YJ, Cho SG, et al. Effect of lifestyle modification on serum chemerin concentration and its association with insulin sensitivity in overweight and obese adults with type 2 diabetes. Clinical Endocrinology 2014;80:825-833.

4.Lee DH, Lee SH, An KY, Moon JY, Kim SH, Choi YJ, et al. Effects of 6 weeks of lifestyle modification including combined exercise program on the risk of metabolic parameters and macrovascular complications in type 2 diabetic patients. The Korean Journal of Obesity 2011;20:147-159.

5.Paffenbarger RS, Jung DL, Leung RW, Hyde RT. Physical activity and hypertension: an epidemiological view. Annals of Medicine 1991;23:319-327.

6.He D, Xi B, Xue J, Huai P, Zhang M, Li J. Association between leisure time physical activity and metabolic syndrome: a meta-analysis of prospective cohort studies. Endocrine 2014; 46: 231-240.

7.World Health Organization. Global recommendations on physical activity for health. 2010

8.Baert V, Gorus E, Mets T, Geerts C, Bautmans I. Motivators and barriers for physical activity in the oldest old: a systematic review. Ageing Res Rev 2011;10:464-474.

9.Jung KH, Oh YH, Kang EN, Kim JH, Sunwoo D, Oh MA et al. National survey of older Korean 2014, Korean Institute for Health and Social Affairs, 2014 p326-327.

10.Hahn V, Halle M, Schmidt-Trucksäss A, Rathmann W, Meisinger C, Mielck A. Physical activity and the metabolic syndrome in elderly German men and women: results from the population-based KORA survey. Diabetes Care 2009;32:511-513.

11.Strath S, Swartz A, Parker S, Miller N, Cieslik L, Health. Walking and metabolic syndrome in older adults. J Phys Act Health 2007;4:398-411.

12.Peterson MJ, Morey MC, Giuliani C, Pieper CF, Evenson KR, Mercer V, et al. Walking in old age and development of metabolic syndrome: the health, aging, and body composition study. Metab Syndr Relat Disord 2010;8:317-322.

13.Lee J, Lee C, Min J, Kang DW, Kim JY, Yang HI, et al. Development of the Korean Global Physical Activity Questionnaire: reliability and validity study. Glob Health Promot 2019:1757975919854301.

14.An KY, Morielli AR, Kang DW, Friedenreich CM, McKenzie DC, Gelmon K, et al. Effects of exercise dose and type during breast cancer chemotherapy on longer‐term patient‐reported outcomes and health‐related fitness: A randomized controlled trial. Internaional Journal of Cancer 2020;146:150-160.

15.Rennie K, McCarthy N, Yazdgerdi S, Marmot M, Brunner E. Association of the metabolic syndrome with both vigorous and moderate physical activity. International Journal of Epidemiology 2003;32:600-606.

16.Rennie KL, Hemingway H, Kumari M, Brunner E, Malik M, Marmot M. Effects of moderate and vigorous physical activity on heart rate variability in a British study of civil servants. American Journal of Epidemiology 2003;158:135-143.

17.Kim MJ, An KY, Jeon YJ Association of type of physical activity and sedentary time with lower back pain in Korean above 50 years: Korean National Health and Nutrition Examination Survey VI 2014, The Korea Journal of Sports Science 2016;25:1107-1120.

18.Bertrais S, Beyeme‐Ondoua JP, Czernichow S, Galan P, Hercberg S, Oppert JM. Sedentary behaviors, physical activity, and metabolic syndrome in middle‐aged French subjects. Obes Res 2005;13:936-944.

19.Bankoski A, Harris TB, McClain JJ, Brychta RJ, Caserotti P, Chen KY, et al. Sedentary activity associated with metabolic syndrome independent of physical activity. Diabetes Care 2011;34:497-503.

TABLES

| Table 1. Participants characteristics | | | |
| --- | --- | --- | --- |
| Variables | Total (N=1,388) | Men (N=594) | Women (N=794) |
| Age (year) | 72.7±5.0 | 72.6±5.0 | 72.7±5.0 |
| Weight (kg) | 61.0±9.8 | 66.4±9.1 | 57.0±8.3 |
| BMI (kg/m2) | 24.3±3.0 | 24.0±2.8 | 24.5±3.2 |
| Waist circumference (cm) | 85.6±8.9 | 87.8±8.8 | 84.0±8.5 |
| SBP (mm/Hg) | 128.9±17.5 | 127.2±17.4 | 130.2±17.6 |
| DBP (mm/Hg) | 72.7±10.0 | 72.2±10.0 | 73.0±10.0 |
| Glucose (mg/dl) | 107.9±24.8 | 110.1±26.4 | 106.2±23.4 |
| HDL (mg/dl) | 48.0±11.5 | 46.1±10.8 | 49.5±11.8 |
| TG (mg/dl) | 131.5±89.8 | 131.1±78.5 | 131.8±97.4 |
| VPA (min/week) | 4.7±40.8 | 9.7±60.4 | 0.9±12.1 |
| MPA (min/week) | 27.0±93.3 | 41.4±114.4 | 16.3±71.9 |
| Walking (min/week) | 229.5±332.6 | 267.7±331.0 | 200.9±331.2 |
| MVPA ≥150min/week (n (%)) | 112 (8.1) | 78 (13.1) | 34 (4.3) |
| Walking ≥180min/week (n (%)) | 603 (43.4) | 295 (49.7) | 308 (38.8) |
| Values are mean±SD or n (%).  BMI, body mass index; SBP, systolic blood pressure; DBP, diastolic blood pressure; HDL, high-density lipoprotein; TG, triglyceride; VPA, vigorous physical activity; MPA, moderate physical activity; MVPA, moderate-to-vigorous physical activity | | | |

| Table 2. Correlation between different intensities of physical activity and metabolic syndrome components | | | | | | |
| --- | --- | --- | --- | --- | --- | --- |
|  | WC | SBP | DBP | Glucose | HDL | TG |
| VPA | -0.010 | -0.001 | 0.010 | -0.017 | 0.064* | -0.056* |
| MPA | -0.050 | -0.085** | -0.048 | -0.043 | 0.047 | -0.068* |
| MVPA | -0.041 | -0.061* | -0.028 | -0.040 | 0.073** | -0.082** |
| walking | -0.005 | -0.006 | 0.014 | -0.027 | 0.051 | -0.065* |
| ** p<0.01. *p<0.05  Adjusted for age, sex, alcohol consumption, and smoking status  WC, waist circumference; SBP, systolic blood pressure; DBP, diastolic blood pressure; HDL, high-density lipoprotein; TG, triglyceride; VPA, vigorous physical activity; MPA, moderate physical activity; MVPA, moderate-to-vigorous physical activity | | | | | | |

| Table 3. Comparison between MVPA and walking with metabolic syndrome components | | | | | |
| --- | --- | --- | --- | --- | --- |
|  | MVPA low | |  | MVPA high | |
| Variables | Walking low  (n=755) | Walking high  (n=521) |  | Walking low  (n=30) | Walking high  (n=82) |
| WC (cm) | 85.9±0.3 | 85.5±0.4 |  | 83.7±1.6 | 84.1±1.0 |
| SBP (mm/Hg) | 129.1±0.6 | 129.5±0.8 |  | 124.4±3.2 | 125.6±2.0 |
| DBP (mm/Hg) | 72.6±0.4 | 72.9±0.4 |  | 70.4±1.8 | 72.5±1.1 |
| Glucose (mg/dl) | 109.3±0.9 | 106.6±1.1 |  | 103.8±4.5 | 103.6±2.8 |
| HDL (mg/dl) | 47.2±0.4 | 48.9±0.5* |  | 48.4±2.1 | 49.9±1.3* |
| TG (mg/dl) | 137.2±3.3 | 127.2±3.9 |  | 134.0±16.4 | 104.8±10.1*^ |
| Estimated mean ±SE  Adjusted for age, sex, alcohol consumption, and smoking status  *p<0.05 vs. MVPA low/Walking low, ^p<0.05 vs. MVPA low/walking high  WC, waist circumference; SBP, systolic blood pressure; DBP, diastolic blood pressure; HDL, high-density lipoprotein; TG, triglyceride; MVPA, moderate-to-vigorous physical activity | | | | | |

| Table 4. The association between meeting exercise guidelines and meeting diagnosis criteria of metabolic syndrome components | | | | | |
| --- | --- | --- | --- | --- | --- |
|  | MVPA | |  | Walking | |
| Variables  (meeting vs. not meeting diagnosis criteria) | <150min vs. ≥ 150min | |  | < 180min vs. ≥ 180min | |
|  | OR (95% CI) | p |  | OR (95% CI) | p |
| WC  (≥90cm for men/≥80cm for women) | 1.35 (0.89-2.06) | 0.16 |  | 1.10 (0.87-1.38) | 0.44 |
| BP  (SBP≥130mmHg or DBP≥85mmHg) | 1.81 (1.18-2.77) | 0.007 |  | 0.99 (0.80-1.24) | 0.95 |
| Glucose  (>100mg/dl) | 1.35 (0.90-2.04) | 0.15 |  | 1.02 (0.82-1.27) | 0.86 |
| HDL  (<40mg/dl for men/ <50mg/dl for women) | 1.14 (0.72-1.79) | 0.57 |  | 1.32 (1.04-1.66) | 0.021 |
| TG  (≥150mg/dl) | 1.54 (0.94-2.53) | 0.09 |  | 1.17 (0.91-1.50) | 0.22 |
| Metabolic syndrome  (≥3 components) | 1.89 (1.19-3.00) | 0.007 |  | 1.17 (0.93-1.47) | 0.17 |
| Adjusted for age, sex, alcohol consumption, smoking status, walking (for MVPA), and MVPA (for walking)  The number of participants meeting diagnosis criteria is 775 for WC, 660 for BP, 724 for glucose, 607 for HDL, 395 for TG, and 589 for metabolic syndrome out of a total of 1,388.  WC, waist circumference; BP, blood pressure; SBP, systolic blood pressure; DBP, diastolic blood pressure; HDL, high-density lipoprotein; TG, triglyceride; MVPA, moderate-to-vigorous physical activity; OR, odds ratio | | | | | |
